# A Case of Diphtheria with Convulsions Cured

**Published:** 1888-05

**Authors:** William H. Krause


### A CASE OF DIPHTHERIA WITH CONVULSIONS CURED.

WILLIAM H. KRAUSE, M. D.

(Read before the Homœopathic Medical Society of the County of New York, March 8th, 1888.)

In the evening of June 12th, 1882, I was called to see a young lady eighteen years old with convulsions. Before I arrived the family had called another physician, who administered a subcutaneous injection of (I suppose) Morphine and left, promising to call if necessary. When I came to the bedside the patient was vomiting, I suppose from the injection, but she had no more convulsions at that time. I found at my examination on both tonsils a dirty, gray, diphtheritic membrane, with all the usual symptoms, fever, headache, pain in bones, etc. I prescribed Merc-cyanide, in water, a teaspoonful every hour. Next morning I found the patient somewhat better as far as the diphtheria was concerned, but the convulsive element of the case had appeared again in full force. With quick jerks the knees would be drawn up, and the head thrust down between them, so that the whole body looked like a bundle. These movements were repeated five or six times in quick succession, followed by a short period of rest, and then more of the same movements. They were so violent that it seemed as though the spinal column must break; but the patient suffered no pain and remained conscious. After five days the diphtheritic membrane had disappeared, but not the convulsive motion. They were worse if anybody came to her bed, and especially if any attempt was made to shake hands with her.

I gave, without success, Ignatia$^{30}$, Stramonium, Hyoscyamus, and Belladonna. I then saw Dr. E. Bayard, and related the symptoms to him. He said, "Belladonna will cure the case." I told him that I had given the 1 and 30th of that without benefit. He said, "Give Belladonna$^{60m}$." I told him I did not have it, so he gave me a powder of Fincke's 60M Belladonna, and told me to give it dry on the tongue and no more medicine for two days, then if all the symptoms had not disappeared to give one dose of Lachesis, which would surely remove the rest of the symptoms. In the afternoon of the sixth day, just after a paroxysm, I gave her the powder on the tongue, and waited for twenty minutes in the room. When I left, I went to the bed and shook the lady's hand for good-bye, and this was the first time that she did not repeat her motion.

There was no subsequent attack. Two days after the patient was discharged cured and able to be about the house.

